# Optimized Infectivity of the Cell-Free Single-Cycle Human Immunodeficiency Viruses Type 1 (HIV-1) and Its Restriction by Host Cells

**DOI:** 10.1371/journal.pone.0067170

**Published:** 2013-06-18

**Authors:** Jin H. Kim, Hanna Song, Jamie L. Austin, Wei Cheng

**Affiliations:** Department of Pharmaceutical Sciences, College of Pharmacy, University of Michigan, Ann Arbor, Michigan, United States of America; Lady Davis Institute for Medical Research, Canada

## Abstract

The infectivity of retroviruses such as HIV-1 in plasma or cultured media is less than 0.1% in general, the mechanisms of which are not yet fully understood. One possible explanation among others is the potential presence of large numbers of defective virions in a virus pool, which limits the apparent infectivity of HIV virions. To test this hypothesis, we have varied the culture conditions used to generate single-cycle HIV-1 virions. Among these culture variables, virion harvest time, media change after transfection, and envelope plasmid input can all improve HIV-1 infectivity by reducing the number of defective virions. A harvest time of 18–24 hours post transfection as opposed to 48 hours, and a media change six hours post transfection both improve viral infectivity. An optimal quantity of envelope plasmid input during transfection was also found. Collectively, these conditions increased the infectivity of HIV-1 virions by sevenfold compared to normally reported values in TZM-bl indicator cell lines. These conditions also increased the infectivity of HIV-1 in CD4^+^ T cells, suggesting that these conditions work by increasing the intrinsic infectivity of a virus pool. Nevertheless, these improvements on virion infectivity were marginal compared to the impact of host cells on HIV infection, which can decrease the apparent infectivity by 19-fold even for the most optimized viruses. These results suggest that the infectivity of HIV-1 virions can be optimized by reducing the number of defective virions; however, viral-cell interactions may pose a major barrier for HIV-1 infectivity.

## Introduction

Compared to many other viruses, the infectivity of cell-free HIV-1 virions is very low. Less than 0.1% of viruses in plasma or culture media are infectious [1], [2], [3], [4], [5], [6]. Although a tremendous amount of knowledge has been learned about this virus over the past 30 years [7], [8], [9], [10], [11], [12], [13], the molecular mechanisms that underlie this apparent low infectivity are still incompletely understood. Broadly defined, two different mechanisms have been proposed to explain this phenomenon. One postulates that a large proportion of virions are inherently defective, with only a small portion of virions highly infectious. In other words, the average infectivity of a virus pool is low due to the presence of defective virions. Alternatively, virions are intrinsically infectious but the viral-cell interactions pose a major barrier for HIV-1 infection, which limits the apparent infectivity of HIV-1 virions. In general, these viral-cell interactions range from initial receptor engagement to provirus integration in the host cell chromosome [7], [8], [13], [14], [15]. Recent evidence has suggested that the attachment of a virus to a host cell or the entry into the cell is a rather inefficient process, which severely limits viral infectivity [6]. Consistent with this view, infectivity of lentivirus preparations based on HIV-1 can be enhanced by association of the virus with magnetic nanoparticles, which facilitates viral attachment to cells through application of a magnetic field [16]. In contrast to these viral entry steps, experiments using HIV-1 pseudo-typed with vesicular stomatitis virus-G envelope revealed a high efficiency for steps post entry; one out of eight virions that initiated reverse transcription could form integrated proviruses [5]. Overall, these studies suggested that HIV-1 virion attachment to host cells is an inefficient process, but once virions gain entry into a host cell, subsequent steps can occur with a relatively high efficiency. This model argues against the presence of defective virions in a virus pool, but supports the idea that HIV-1 virions are intrinsically infectious. Reasonable as they sound, there are caveats in reaching these conclusions. The high infectivity of HIV-1 virions revealed from the above studies was for viruses that were either pre-adsorbed on host cell surface or which had already initiated reverse transcription. In the virus pool, there were still large populations of unadsorbed virions or virions that had not initiated reverse transcription. Whether they are defective virions, i.e., virions that are deficient in receptor engagement or initiation of reverse transcription remains unknown.

Defective virions can arise naturally in the viral life cycle, with one or more genes required for viral replication missing or defective in the virions [17]. This mechanism may operate due to mutations introduced by HIV reverse transcriptase (RT), which has a high error rate during the synthesis of provirus DNA [18], [19], [20], and also the host cell defense mechanisms such as APOBEC3 cytidine deaminases [21], [22], which can introduce hypermutation to the proviral DNA during reverse transcription. The production of defective virions due to mutations contributes to the heterogeneity of a virus pool, which may significantly complicate the study of viral infectivity. Alternatively, molecularly cloned HIV-1 that is capable of only a single round of infection [23], [24] offers a unique tool to address these important questions. The production of these virions in cell culture involves the use of a mutant provirus clone together with a separate plasmid that drives the expression of viral envelope glycoproteins. Because viral proteins are expressed from cloned DNA instead of the provirus reversed transcribed from a RNA genome by RT, mutations in viral proteins that arise from RT errors or APOBEC3 activity are virtually eliminated. By varying culture conditions used to produce these virions, one can potentially optimize the infectivity of HIV-1 and address the intrinsic infectivity of these virions. Moreover, because cells infected by these single-cycle virions result exclusively from the initial input virus, the efficiency of provirus integration can be correlated with the efficiency of viral entry without complications from multiple rounds of infection.

Although single-cycle HIV-1 virions have been widely used for viral neutralization assays [25] and evaluation of antiviral drugs [26], conditions to optimize their infectivity in cell culture have not been extensively reported. The typical procedures use an equal-weight mixture of provirus and envelope plasmids for transfection in 293 or 293T cells, and virions are harvested 48 hours post transfection [23]. Because the expression of provirus and envelope glycoproteins is separate in two plasmids, the ratio between the two plasmids may drive the expression of Gag and envelope glycoproteins at different levels. As a result, the overall infectivity of virions may vary depending on the level of envelope protein incorporation [27], [28]. Aside from the ratio between the two plasmids, the rationale for harvesting virions 48 hours post transfection is not extensively documented. HIV virions are known to undergo inactivation with time due to biophysical instability of virion proteins [1]. This inactivation can occur simultaneously with virion production and thus an optimal time frame for harvesting virion from cell culture may be expected. Indeed, recent studies on replicative HIV-1 virions revealed that virions harvested at early time points from culture media have higher infectivity on a per particle basis [6], suggesting that HIV-1 virion infectivity may be optimized through variation in their culture conditions.

Driven by this idea, we set out to produce single-cycle HIV-1 virions in cell culture and vary culture conditions to optimize the infectivity per virion particle. Among these culture variables, virion harvest time, media change after provirus transfection, and input quantity of envelope plasmid can all impact the infectivity of virions produced. A harvest time of 18–24 hours post transfection as opposed to 48 hours, and a media change six hours post transfection both improve viral infectivity. An optimal quantity of envelope plasmid input was also found. Collectively, these conditions can generate HIV-1 virions with infectivity that is sevenfold higher than normally reported values in TZM-bl indicator cell lines. Our data suggest that the increase in virion infectivity results from the reduction of defective virions in a virus pool. Consistent with this view, the above conditions also increased the infectivity of HIV-1 virions in CD4^+^ T cells, suggesting that these conditions work by increasing the intrinsic infectivity of a virus pool. Nevertheless, these improvements for virion infectivity were marginal compared to the impact of host cells on virion infectivity. Infectivity for the same batch of optimized virions can vary by 19-fold depending on the host cell lines used. These results indicate that the infectivity of HIV-1 virions can be optimized through variation of culture conditions, which works by reducing the number of defective virions in a virus pool. However, the difference in viral-cell interactions may pose a major barrier that restricts HIV-1 virion infectivity [29], [30].

## Results

### Measurement of Infectious Particle Concentration for Single-Cycle Viruses

**Figure 1:**
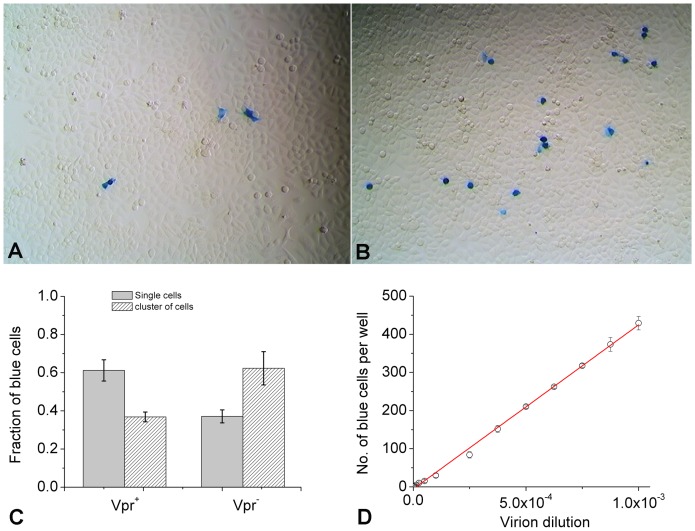
Measurement of infectious HIV-1 virions by blue-cell counting in TZM-bl cells. (A) and (B), representative images of blue TZM-bl cells in 12-well plates for quantitation of infectious particles showing well-resolved single cells and clusters of cells such as doublets. (C) Fraction of single or clustered blue cells depends on the presence of Vpr in single-cycle HIV-1 virions. Vpr^+^ virions were generated from transfection of 293T cells with 1 μg pNL4-3E^-^ and 0.1 μg pcDNA3.1REC. Vpr^−^ virions were generated from transfection of 293T cells with 1 μg pNL4-3R^−^E^−^ and 0.1 μg pcDNA3.1REC. Both virions were harvested 48 hours post transfection without media change. TZM-bl cells were infected by either of these two virions and the resulting single or clustered blue cells were counted. Error bars are standard deviations from six independent replicates of the same experiments. (D) The number of blue cells was plotted as a function of virion dilution factor per 100 μl of virus. The red line shows its linear regression. The virus was produced by transfection of 293T cells using 1.0 μg pNL4-3E^−^ plasmid and 0.2 μg pcDNA3.1REC, followed by collection 48 hours post transfection without media change. Error bars are standard deviations from six independent replicates of the same experiments.

Throughout this work, we define infectivity as the fraction of virions that can establish a productive infection in a host indicator cell line. To establish a quantitative framework for measurement of infectivity, we generated single-cycle virions using NL4-3 virus and infected TZM-bl cell lines [31] using virions produced under various conditions. This cell line has been one of the popular cell lines for HIV-1 infection and thus comparison among labs is possible [32], [33], [34], [35]. Cells were infected for two hours at 37°C and further incubated for 48 hours to allow production of β-galactosidase in infected cells. We then used X-Gal staining to visualize these infected cells, which appeared in blue color [36]. It is worth noting that un-integrated provirus DNA can also turn on *tat* transcription [37] and cells may turn blue in the absence of the provirus integration [38]. However, this integration-independent infectivity is only 5% of total infectivity observed for NL4-3 virus in TZM-Bl cells [39]. Thus, counting stained cells under a microscope (Fig. 1A, B) measures the number of cells that have been productively infected by NL4-3 virus, i.e., provirus has been integrated into cellular chromosomes, which upon transcriptional activation, produces Tat protein that subsequently activates the expression of β-galactosidase. In the presence of viral Vpr protein [40], [41], [42], [43], majorities of blue cells were singles and each blue cell was counted as resulting from one infectious particle under current Multiplicity of Infection (MOI) conditions (<0.005). Occasionally, blue cells appeared as clusters due to cell division and the cluster of blue cells was counted as resulting from one infectious particle. This counting method was supported by our observations shown in Fig. 1C, where the number of clustered blue cells was correlated with the progression of cell cycle. HIV-1 virions carrying Vpr proteins resulted in less clusters of blue cells due to cell cycle arrest by Vpr proteins [40], [41], [42], [43]. To examine the linearity of this assay, we varied the dilution factors for the input virions and the resulting blue cells were counted. A typical result is shown in Fig. 1D, where the number of blue cells was plotted as a function of the dilution factor for each 100 μl of virus. Linear regression of this plot yields a straight line with an intercept close to zero (−5.4±2.5) and a slope of (4.30±0.05) ×10^6^/ml with adjusted R-square of 0.998. Because the multiplicities of infection (MOI) were less than 1 under these conditions (the highest MOI  = 0.005), this dependence strongly suggests that a single HIV-1 virion is capable of establishing an infection, and each blue cell resulted from infection by a single virus, with 95% chance of being an integrated provirus. In fact, for even the most concentrated virus in these experiments, the total number of viral physical particles as determined using p24 ELISA was less than the total number of starting cells. Thus, the slope in Fig. 1D directly measures the concentration of infectious particles, C_i.p._.

**Figure 2:**
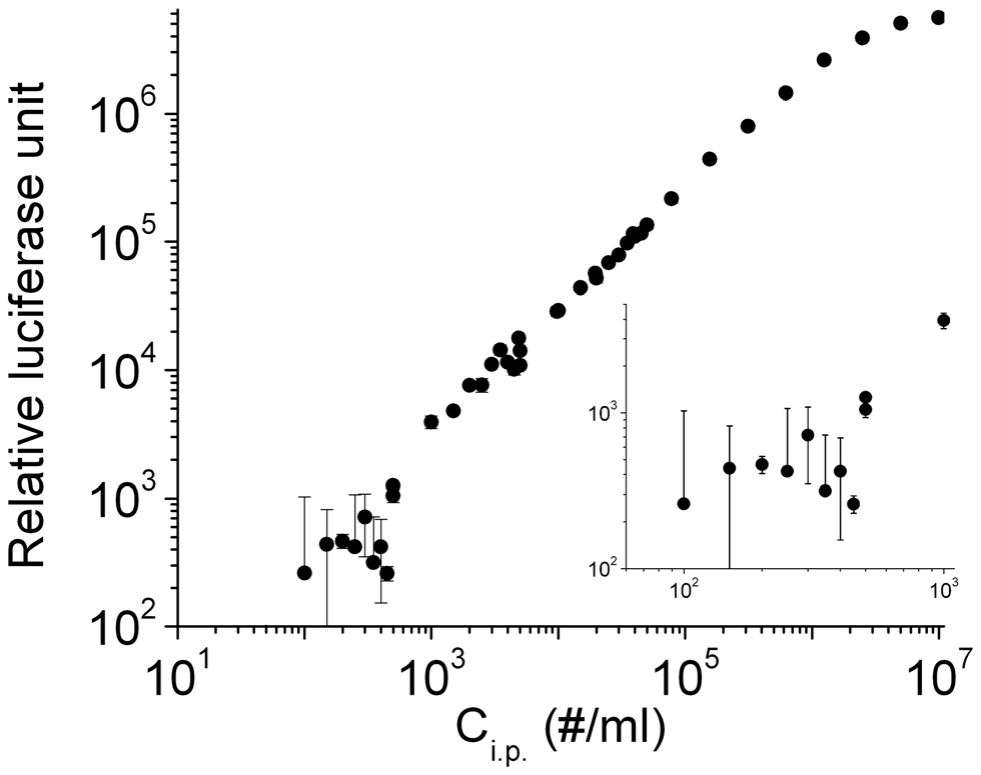
Luciferase activity assay to monitor infection of TZM-bl cells by single-cycle HIV-1 virions. The RLUs measured with a microplate reader were plotted as a function of input C_i.p._ on a double-logarithmic scale. It shows saturation behavior when C_i.p._ exceeds 10^6^/ml, and shows excess noise when C_i.p._ is below 10^3^/ml (inset). Error bars are standard deviations from three independent replicates of the same experiments.

Because the TZM-bl cell line carries luciferase reporter, we also tested a luciferase assay using virions that had been quantitated using blue cell counts. As shown in Fig. 2, the relative luciferase activity measured by the plate reader is largely linear as a function of input virion concentrations. However, when C_i.p._ falls below 1000/ml, the data are too noisy to determine C_i.p._ of the input virion accurately (inset). This noise is in part due to TZM-bl cells alone that have a background as high as 6,000 Relative Luciferase Unit (RLU) in the absence of any virion infection. Compared to the luciferase assay, the blue-cell counting method has a lower background and higher sensitivity. Indeed, the blue-cell assay allows us to measure titer as low as one infectious particle per infection. Infected cells are counted at single-cell resolution, which is not possible for the luciferase assay due to variation of enzyme activity across different cells [36], [44]. For all these reasons, we have used blue-cell counting to determine C_i.p._ in TZM-bl cells throughout, and compare them for virions produced under various conditions.

### Measurement of Physical Particle Concentration for Single-Cycle Viruses

**Figure 3:**
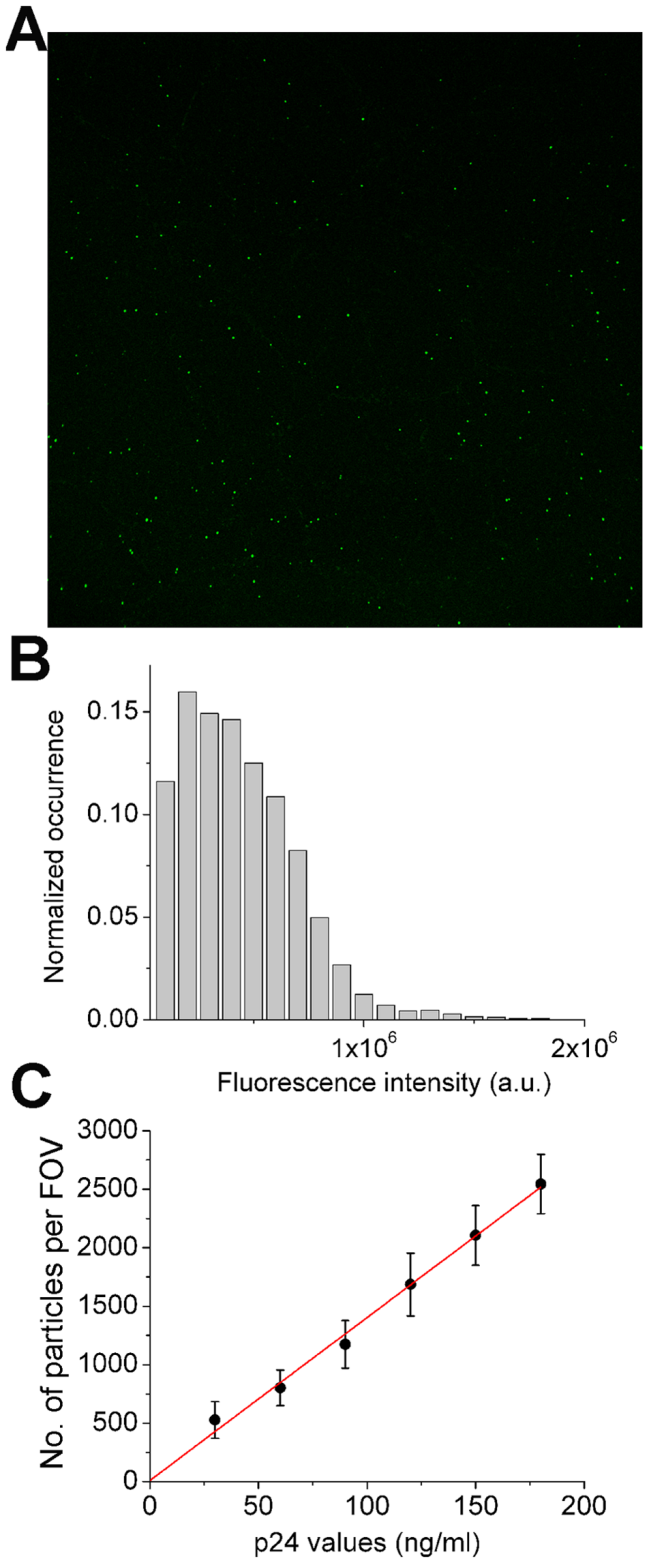
Confocal imaging of EGFP-Vpr virions and particle quantitation. (A) A representative fluorescence image of EGFP-Vpr virions deposited on a coverslip surface. The image is 1024 by 1024 pixels with a physical size of 124 by 122 nm in x and y dimensions. (B) Distribution of virion fluorescence intensity identified from the confocal image using a custom-written MATLAB script. (C) The correlation between numbers of fluorescent particles identified from a FOV and the input p24 values measured using ELISA. The linear regression is shown in red line. Error bars for each sample are standard deviations from at least nine different areas analyzed on a coverslip surface.

The stoichiometry of Gag protein in HIV-1 has been determined to be approximately 2,500 per virion [45], [46]. Using a molecular weight of 24 kD for p24, this yields 1×10^7^ particles per ng of p24. One can thus estimate the number of physical particles based on p24 ELISA measurement. To support this approach for measurement of C_p.p._, the concentration of physical particles, we have developed a confocal imaging and quantitation procedure to count the number of virions and examine its correlation with p24 values. This procedure uses virions labeled with enhanced green fluorescent proteins (EGFP) fused to Vpr, which allows direct visualization of virions encapsulating EGFP-Vpr under a confocal fluorescence microscope (Fig. 3A). To objectively quantitate the number of virions in a field of view (FOV), we developed a custom-written MATLAB script, which could identify fluorescent particles automatically and also output fluorescence intensity associated with each particle for statistical purposes. Fig. 3B shows a representative fluorescence intensity histogram associated with particles identified. To examine the correlation between the number of particles and p24 measurement, EGFP-Vpr virions with various p24 values were deposited onto PLL-coated coverslips and the resulting particle numbers in a FOV were measured as described in Experimental Procedures. Fig. 3C plots one such result, where the number of fluorescent particles identified in a FOV is linearly correlated with their p24 values, with adjusted R-square of 0.992 and intercept on y-axis close to zero. Although this imaging method cannot provide a direct conversion between the number of virion particles and p24 values due to the potential presence of virions without EGFP, this correlation supports p24 values as a measurement of viral C_p.p._.

### Harvest Time Dependence of Virion Infectivity

**Figure 4:**
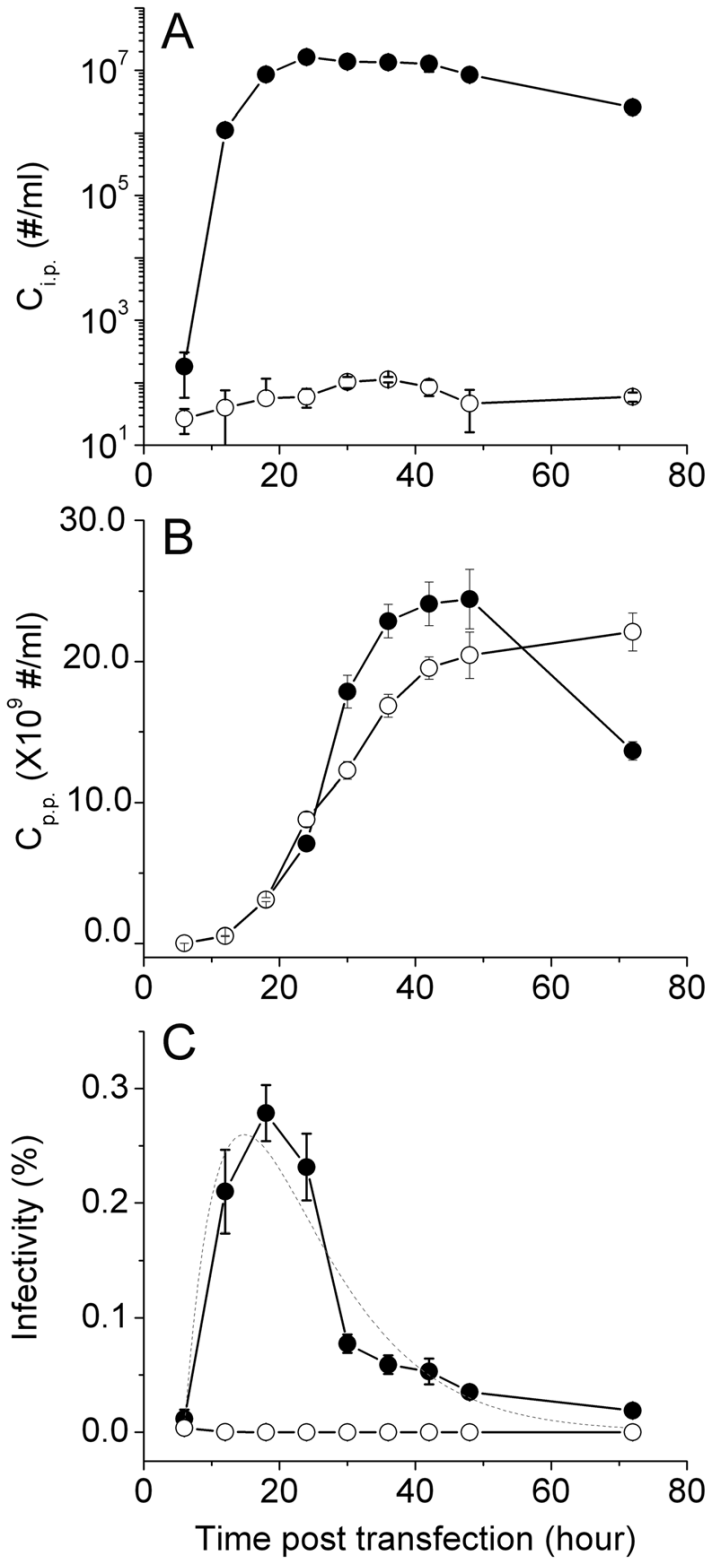
C_i.p._, C_p.p._, and infectivity of HIV-1 virions as a function of harvest time post transfection. Filled circles are for virions produced using 1.0 μg pNL4-3E^−^ plasmid and 0.2 μg pcDNA3.1REC. Open circles are for Gag particles produced using 1.0 μg pNL4-3E^−^ plasmid only. The dashed line is (C) is fit of the time course to a sum of two-exponential kinetics. Error bars are standard deviations from three independent replicates of the same experiments.

Recent studies on replicative HIV-1 virions revealed that virions harvested at early times from culture media have higher infectivity [6]. To explore this phenomenon for single-cycle HIV virions, we transfected 293T cells with a pNL4-3E^−^ plasmid encoding the mutant provirus together with an envelope plasmid pcDNA3.1REC encoding NL4-3 envelope and collected HIV-1 virions at different times post transfection. For each collection, we determined C_i.p._ by blue-cell counting and measured C_p.p._ by p24 ELISA. The infectivity of virions was then calculated as the ratio between these two concentrations. As shown in Fig. 4A, infectious particles can be detected as early as six hours post transfection (filled circles), reaching a peak value around 24 hours and the C_i.p._ starts to drop off slowly afterwards. In contrast, the concentration of physical particles C_p.p._ continues to increase until 48 hours and then drops off (Fig. 4B). As a result, the infectivity of virions collected at different times shows a pronounced dependence on harvest time, reaching a maximum at 18 hours and dropping off thereafter (Fig. 4C). The difference in infectivity can be as large as fivefold for virions collected at different time points post transfection. This time dependence has interesting implications for the production of HIV-1 virions by 293T cells; either the virions produced at early times lose infectivity with time, or more defective virions are produced at later times after transfection.

**Figure 5:**
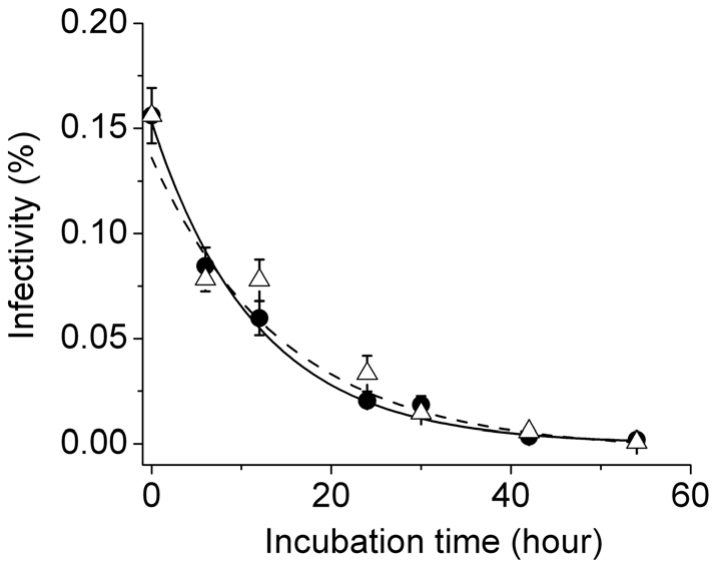
Kinetics of HIV-1 virion infectivity decay. Time course of HIV-1 virion spontaneous inactivation was measured in either 12-well plate (triangle) and fitted using single exponential decay (dashed line), or in 1.5 ml test tube (circles) and fitted using single exponential decay (solid line). Error bars are standard deviations from three independent replicates of the same experiments.

The infectivity decay of HIV-1 virions has been reported previously [1], which is attributable to loss of reverse transcriptase activity, biophysical instability of virion particles and possibly gp120 shedding, although the shedding of gp120 was later shown to be insignificant for NL4-3 virus [47]. The decay of HIV-1 virion infectivity in Fig. 4C has a half life of 6.6 hours or greater as determined by fitting the infectivity time course to a two-exponential kinetic model (dashed line). To examine the cause of this infectivity decay, we measured the apparent rate of virion inactivation. These experiments were done by incubating the harvested HIV virions at 37°C in either 12-well plates or 1.5-ml polypropylene test tubes. After various time of incubation, aliquots of virions were taken and assayed for infectivity. The dependence of virion infectivity as a function of incubation time is plotted in Fig. 5. For time courses measured in 12-well plates (triangle), it can be well fit by a single exponential model (dashed line) with a relaxation time of 14.7±2.3 hours; for time courses measured in test tubes (filled circle), it can be well fit by single exponential (solid line) with a relaxation time of 11.7±0.9 hours. The similar rates of infectivity decay from these two measurements suggest that both measure the apparent rate of virion inactivation, which may also contain contributions from container-specific virion adsorption. These relaxation times define a half-life of infectivity decay between eight and ten hours at 37°C in complete media, which is slower than the rate of infectivity decay during virion production. This quantitative comparison suggests that the infectivity decay in Fig. 4C is not solely due to spontaneous inactivation of these virions in culture media, but rather, production of defective or less infectious virions occurs at the same time so that the overall rate of infectivity decay speeds up. Additional evidence for the presence of defective virions was revealed from further experiments that we will describe below.

### Media Change Post Transfection Increases HIV-1 Infectivity

**Figure 6:**
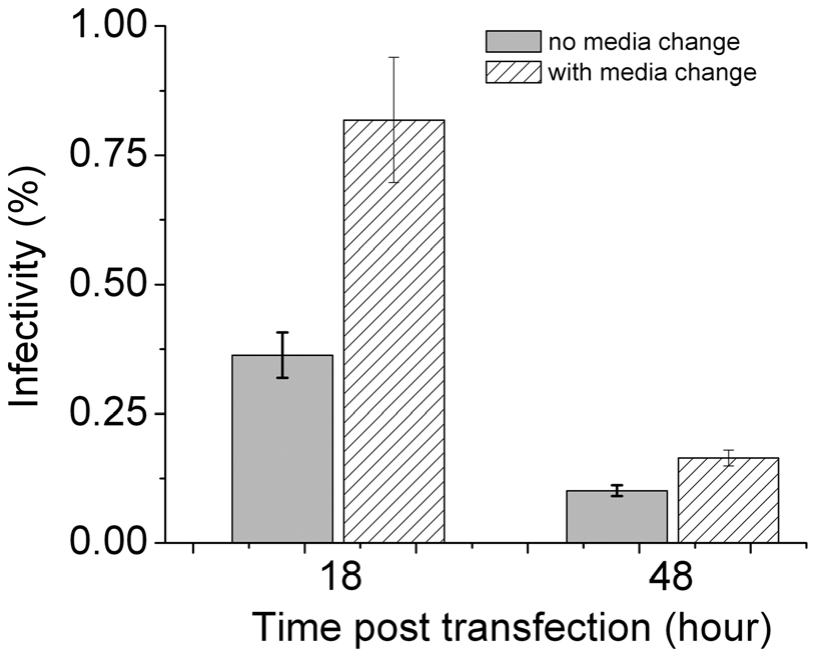
Effect of media change post transfection on HIV-1 virion infectivity. The virus was produced by transfection of 293T cells using 1.0 μg pNL4-3E^−^ plasmid and 0.2 μg pcDNA3.1REC, and virions were collected at 18 and 48 hours post transfection. The differences in infectivity were confirmed by the Paired-sample t-test. Error bars are standard deviations from five independent replicates of the same experiments.

**Figure 7:**
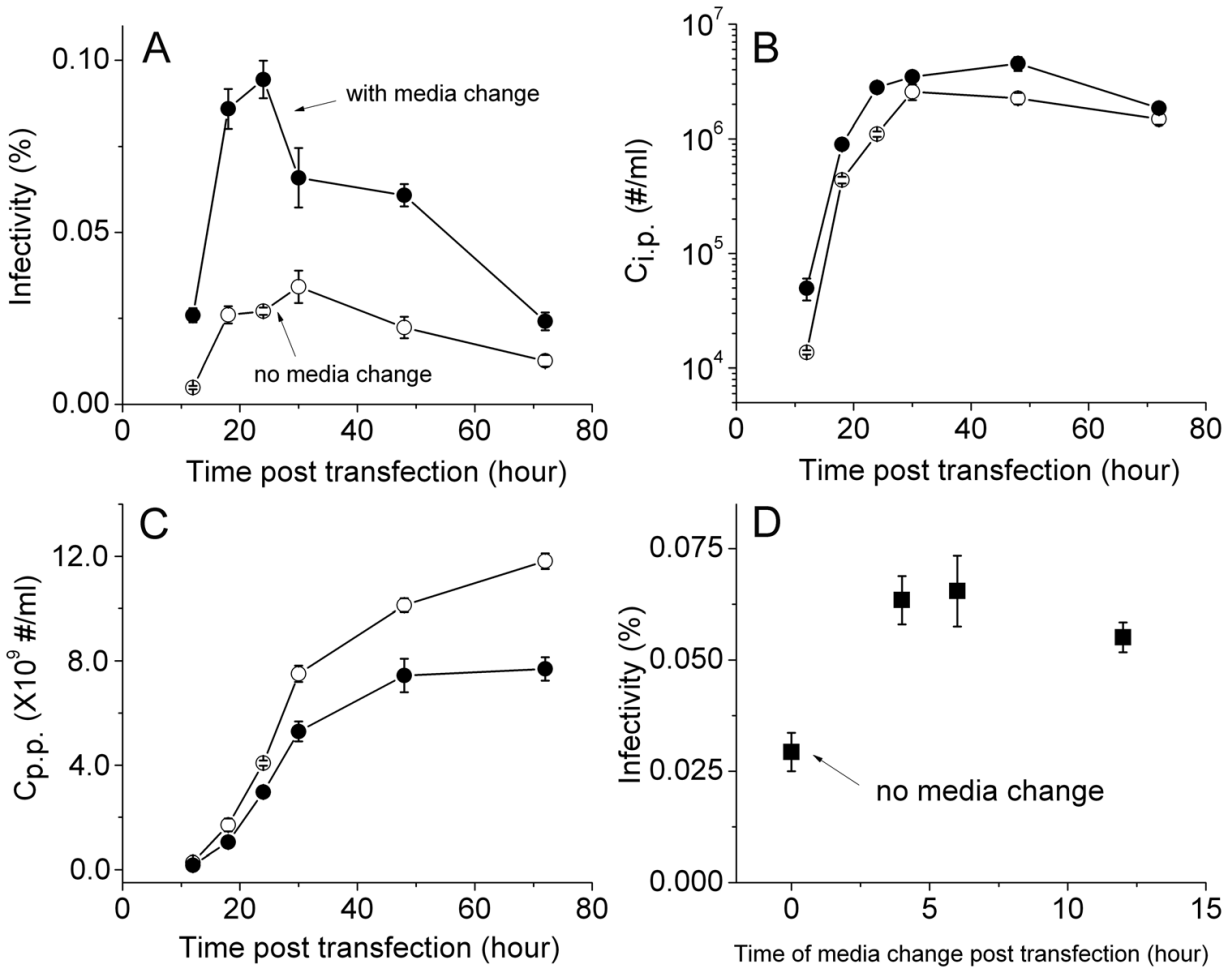
Effect of media change on HIV-1 virion infectivity tagged with EGFPs. Panels (A), (B) and (C) show the infectivity, C_i.p._ and C_p.p._ of HIV-1 virions tagged with EGFP-Vpr, as a function of harvest time post transfection, with media change six hours post transfection (filled circles) and without (open circles). Panel (D) shows the infectivity of free EGFP virions as a function of the time of media change post transfection. The virus was collected 24 hours post transfection. Error bars are standard deviations from three independent replicates of the same experiments.

**Figure 8:**
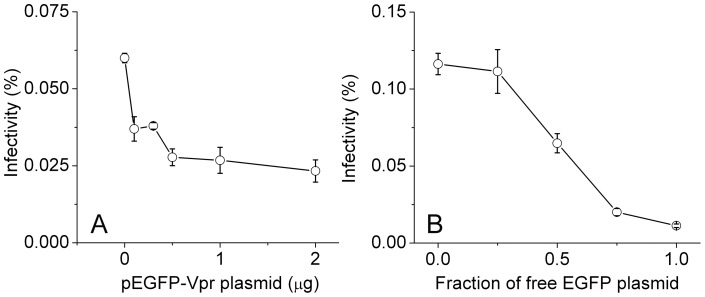
Infectivity of EGFP-tagged single-cycle virions as a function of input EGFP. (A) Infectivity of EGFP-Vpr virions as a function of input pEGFP-Vpr plasmid. The viruses were collected 48 hours post transfection without media change. (B) Infectivity of free EGFP virions as a function of the fraction of EGFP-tagged mutant provirus in a total of 1 μg mutant provirus. The virions were collected 24 hours post transfection without media change. Error bars are standard deviations from three independent replicates of the same experiments.

The transfection reagent we used, *Trans*IT-LT1, is a low toxicity reagent that is comprised of a lipid and protein/polyamine mixture. Although media change is not required after transfection using this reagent, we decided to replace the media to test if infectivity of HIV-1 virions changes. As shown in Fig. 6, media change has a positive impact on virion infectivity. The infectivity of HIV virions collected 18 hours post transfection increased around twofold with media change at six hours post transfection (p-value 6.943×10^−4^ from paired-sample t-test), reaching infectivity close to 1%. This enhancement is also true for virions harvested 48 hours post transfection (p-value 2.054×10^−4^ from paired-sample t-test), although not as pronounced as 18-hour virions. This effect of media change was also confirmed in two other single-cycle HIV-1 virions: virions tagged with EGFP through Vpr-fusion [48], and virions tagged with free EGFP [49]. As shown in Fig. 7A, the infectivity of EGFP-Vpr virions at early harvest times is higher than later ones, similar to the untagged virus. Notably, the infectivity of virions generated with media change six hours post transfection (filled circles) is two or threefold higher than those without (open circles). This increase in infectivity is due to a slight increase in C_i.p._ and a concomitant decrease in C_p.p._, as shown in Fig. 7B and C. Moreover, for free EGFP virions, the time of media change is optimal at six hours post transfection, as shown in Fig. 7D, where the infectivity of virions is plotted as a function of time at which the media was changed. These results confirm the findings we made for the untagged virions and show that media change after transfection increases HIV-1 virion infectivity. We noticed, however, that the infectivity for both EGFP-tagged virions is lower than those untagged viruses, likely due to the inhibitory effect of EGFP tagging on HIV-1 virion infectivity. This effect of inhibition is clear from Fig. 8A, where we varied the input pEGFP-Vpr plasmid systematically and measured the infectivity of resulting virions. A similar trend of inhibition was also observed for free EGFP virions, where an increasing fraction of EGFP-tagged mutant provirus decreases virion infectivity (Fig. 8B).

**Figure 9:**
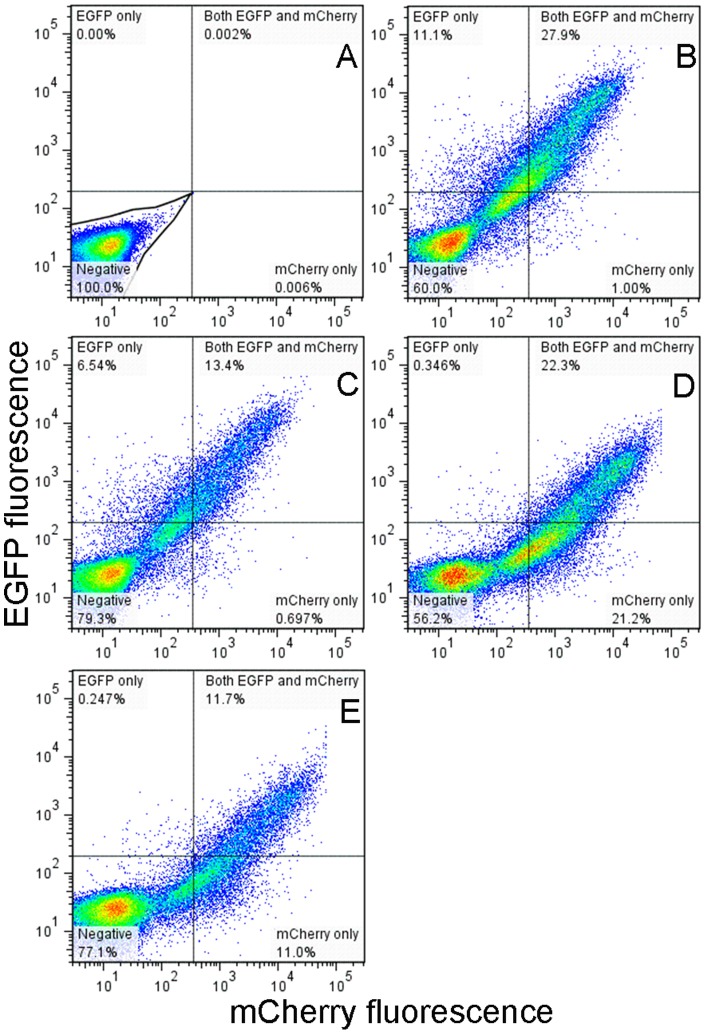
Two-color flow cytometry to quantitate fraction of cells that carried reporter plasmids. (A) Negative control in the absence of both plasmids, which was used to gate cell populations in transfected cells. (B) 293T cells transfected with 0.6 μg pEGFP-Vpr and 0.2 μg pmCherry-Vpr in 2-ml volume in a 35-mm dish. The gates established based on negative control were used to quantitate fractions of cells that carry EGFP only, mCherry only and both fluorescent proteins in a mixed populations, with the percentages of each population indicated in the cytogram. (C) 293T cells transfected with 0.6 μg pEGFP-Vpr and 0.2 μg pmCherry-Vpr, with a media change six hours post transfection; (D) 293T cells transfected with 0.2 μg pEGFP-Vpr and 0.6 μg pmCherry-Vpr; (E) 293T cells transfected with 0.2 μg pEGFP-Vpr and 0.6 μg pmCherry-Vpr, with a media change six hours post transfection. All cell samples were collected at 48 hours post transfection. 488 nm and 560 nm lasers were used for excitation of EGFP and mCherry respectively. Two independent analyses yielded results that were identical within errors.

Consistently, we have observed that changing media post transfection benefits HIV-1 infectivity. What is the mechanism behind the effect of media change on HIV virion infectivity? It is possible that this increase in infectivity results from better environmental conditions for the transfected cells. In fact, typical transfection procedures such as calcium phosphate transfection require media change from 4 to 16 hours post transfection in order to maintain healthy cell growth [50], [51]. However, our data reliably demonstrate that increases in C_i.p_ and concomitant decreases in C_p.p._ led to an overall increase in infectivity for all of the different virions we have produced. This phenomenon indicates that media change post transfection may remove potential sources of defective virions. Where do these defective virions come from? At six hours post transfection, the level of Gag production is still very low (Fig. 4). The observed decrease in C_p.p._ due to media change is more than the amount of C_p.p._ produced at six hours post transfection, suggesting that these defective virions are actually produced more than six hours after transfection. Gag particles that do not carry any envelope proteins may be one potential source of defective virions, which may be produced through two different mechanisms under current conditions. First, even though the two plasmids were transfected simultaneously during the production of single-cycle virions, some cells may receive only the mutant provirus instead of the two plasmids. We initially produced virions using 1 μg mutant provirus DNA (14.8 kb) and 0.2 μg envelope plasmid (8.6 kb) (Fig. 4). This amount of envelope plasmid was established during our initial optimization for virions collected 48 hours post transfection. Under these conditions, mutant provirus DNA is in ∼ threefold molar excess over the envelope plasmid, which may favor the uptake of mutant provirus only. To test this hypothesis, we transfected 293T cells with a mixture of two plasmids at different molar ratios, pEGFP-Vpr and pmCherry-Vpr. Because these two plasmids carry the same promoters and are identical in sequence other than the fluorescent protein reporter, the difference in reporter protein expression can be directly related to the partition of these two plasmids in different cells. The distinct colors of EGFP and mCherry allow us to quantitate their expression at single cell level using flow cytometry. These results are shown in Fig. 9, with mock-transfected cells to set the gates for quantitation of EGFP or mCherry positive cells (Fig. 9A). When the amount of pEGFP-Vpr was in threefold molar excess over pmCherry-Vpr (Fig. 9B), a significant fraction of cells (11.1%) had low or undetectable mCherry fluorescence. In contrast, 27.9% of cells carried both plasmids as indicated by the presence of two colors in a single cell above fluorescence thresholds. Upon media change, the fraction of cells that showed low or undetectable mCherry fluorescence decreased by 41% ((11.1–6.54)/11.1×100%, Fig. 9C). Conversely, when the amount of pmCherry-Vpr was in threefold molar excess over pEGFP-Vpr, 21.2% of cells had low or undetectable EGFP fluorescence, in contrast to 22.3% of cells that carried both plasmids (Fig. 9D). Upon media change, the fraction of cells that showed low or undetectable EGFP fluorescence decreased by 48% ((21.2–11.0)/21.2×100%, Fig. 9E). These results suggest that depending on the ratio between the two plasmids, a significant number of cells may only receive one of the plasmids after transfection. Even though all cells may have received both plasmids, these two plasmids are at such a great disproportion that expression from one of them is low or undetectable. This mechanism will allow 293T cells to uptake only the mutant provirus DNA and thus give rise to the production of defective Gag particles. Alternatively, both plasmids are taken up by the cells but envelope DNA is very low, which may give rise to less infectious virions. To test whether uptake of the mutant provirus alone can generate Gag particles under our conditions, we transfected 293T cells with just the mutant provirus DNA and assayed the amount of Gag proteins secreted into culture media using p24 ELISA. The time course of Gag particle production is shown in Fig. 4B as open circles, which displays a time dependent increase that is very similar to the time course of virion production. Infection assays using TZM-bl cells revealed negligible numbers of blue cells (open circles in Fig. 4A) and the resulting infectivity for these particles was five orders of magnitude lower than enveloped virions (Fig. 4C). Taken together, the above results suggest that there is a mechanism for production of defective particles during the production of single-cycle virions. Cells that receive the mutant provirus only will generate Gag particles without envelope glycoproteins, which reduce the overall infectivity of HIV-1 virions. Media change after transfection may lower the fraction of these defective or less infectious virions and improve the overall infectivity of HIV-1 virions. However, how this mechanism works quantitatively remains to be determined. The second mechanism that can generate Gag particles without envelope glycoproteins is the kinetics of envelope protein incorporation. It is known that HIV envelope proteins must undergo extensive post-translational modifications to arrive at plasma membrane for incorporation into virions [52]. If this process occurs more slowly than Gag assembly and budding, then during the early phase of virion production, Gag particles could be produced without envelope proteins. Future study is necessary to test this hypothesis.

### Dependence of HIV Infectivity on Envelope Plasmid Input

**Figure 10:**
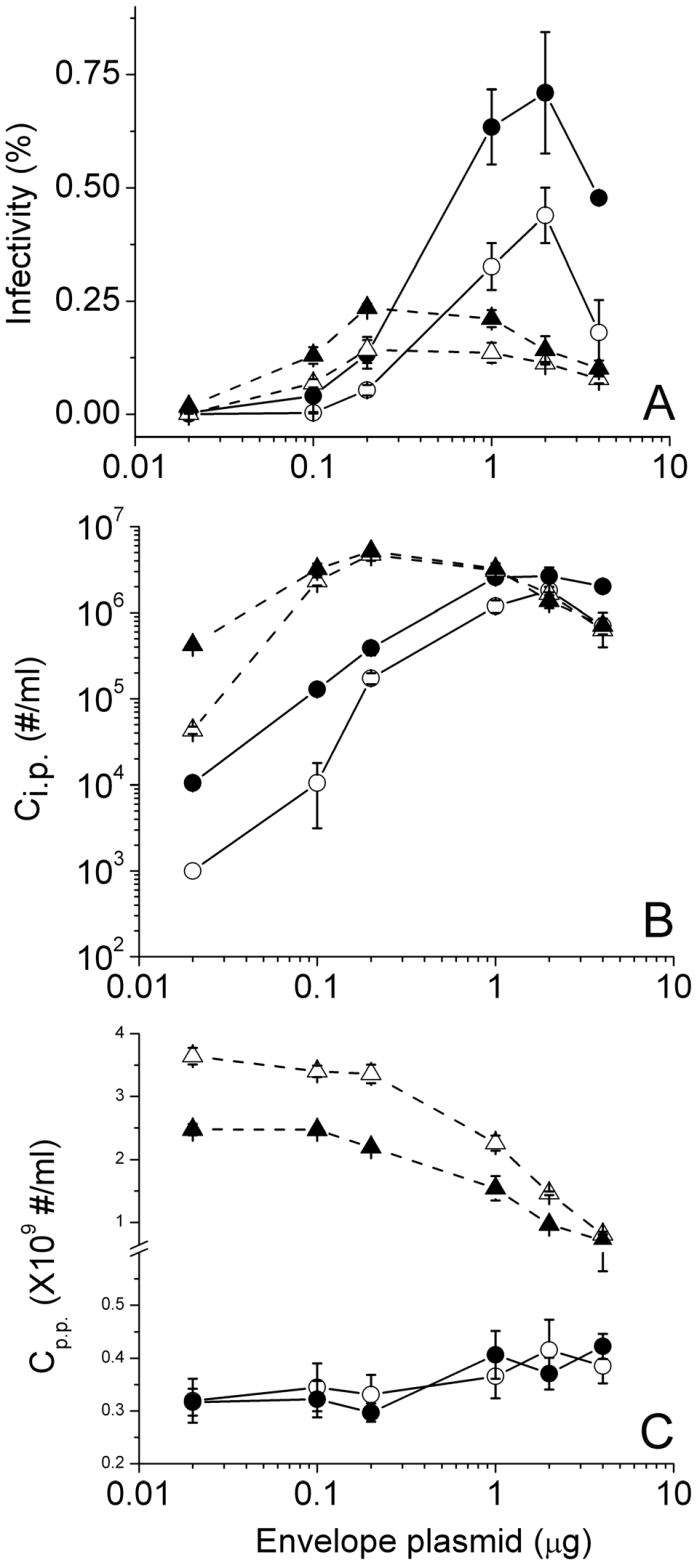
Dependence of HIV-1 virion infectivity on envelope plasmid input during transfection. (A), (B) and (C) show the infectivity, C_i.p._ and C_p.p._ for virions produced with various inputs of envelope plasmid pcDNA3.1REC. Circles and triangles are for virions collected 18 and 48 hours post transfection, respectively, with filled symbols representing samples with media change and open symbols representing samples without media change. Error bars are standard deviations from three independent replicates of the same experiments, except for 48 hours without media change (open triangle dashed line), which were duplicates.

**Figure 11:**
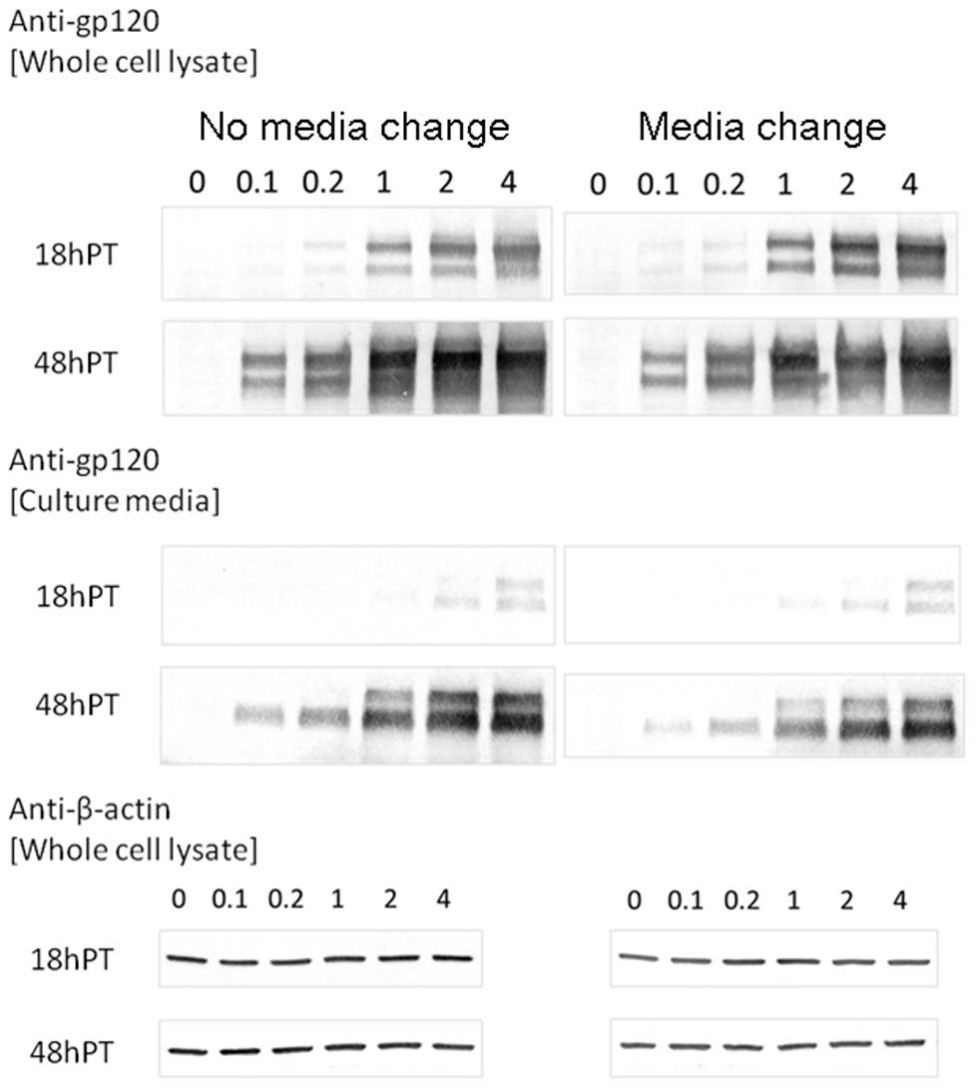
Western blotting of virion and cell lysate samples collected from cells transfected under various conditions. The amount of envelope plasmid in μg is indicated above the lanes. The double bands from anti-gp120 staining corresponded to gp120 (bottom) and gp160 (top), respectively. β-actin expression was also probed simultaneously to validate the comparison across different transfection conditions. Two independent analyses yielded results that support the same conclusions.

The above experiments suggest that by varying the ratio between the two plasmids used in transfection, more envelope plasmid may be taken up by cells that can produce virions of higher infectivity. To test this hypothesis, we produced virions by transfecting 293T cells with a mixture of the two plasmids at various input ratios. In these experiments, a constant mutant provirus DNA (1 μg) was used throughout but the envelope plasmid was varied from 0.02 to 4 μg. Thus, the molar ratio between the two plasmids varied over two orders of magnitude. To equalize transfection conditions, we also included pcDNA3.1 vector DNA so that total DNA input was the same for all the transfections (5 μg). Virions were collected at two different time points post transfection, 18 and 48 hours, with and without media change at six hours post transfection. We then determined their infectivity using blue-cell counting and p24 ELISA assay. The results are shown in Fig. 10, where filled symbols stand for media change and open symbols for no media change, solid lines for virions collected 18 hours post transfection and dashed lines for virions collected 48 hours post transfection throughout. As shown in Fig. 10A, for both 18 and 48 hours, virion infectivity with media change is generally higher than those without, consistent with previous findings (Fig. 6 and 7). Notably, the infectivity of HIV virions changes with increasing envelope plasmid. For virions collected 18 hours post transfection, the infectivity increased to a peak value of 0.7% at 2 μg envelope but declined at 4 μg envelope. This increase in infectivity resulted from a steady increase of infectious particles secreted into the culture media (C_i.p._ in Fig. 10B) but without much change in the number of physical particles (C_p.p._ in Fig. 10C), suggesting that particles of higher infectivity were generated. As shown by western blotting of these samples in Fig. 11, more gp120 proteins were produced in 293T cells in response to higher envelope plasmid input. More gp120 proteins were also observed in culture media, suggesting that more envelope glycoproteins might be incorporated into Gag particles which in turn increase the infectivity of HIV-1 virions [27]. We notice, however, that more precursor glycoprotein gp160 was also produced in 293T cells with increasing envelope plasmid. This trend is true for culture media, suggesting that gp160 may be incorporated into virions [53] when these proteins are produced at high concentrations in the cell. Because gp160 is nonfunctional during HIV-1 infection [54], the incorporation of gp160 into virions [53] may underlie the apparent decrease of C_i.p._ and infectivity with high envelope inputs. We emphasize, however, that the above Western results are only suggestive but not conclusive due to the presence of both gp120 and gp160 in culture media upon transfection of 293T cells by envelope only plasmid, which we conducted independently in the absence of provirus transfection. Future experiments are thus necessary to distinguish among various forms of envelope glycoproteins, including gp120 incorporated into virions and gp160 that may be secreted by cells in the form of vesicles.

In contrast to 18-hour virions, infectivity for virions collected 48 hours post transfection increased to a peak value of 0.24% at 0.2 μg envelope and declined thereafter (Fig. 10 dashed lines). The increase of infectivity for up to 0.2 μg envelope DNA resulted from an increase in C_i.p._ (Fig. 10B) but little change in C_p.p._ (Fig. 10C), suggesting that more envelope incorporation into Gag particles increases virion infectivity. Among these observations, we also notice that the infectivity of virions collected 18 hours post transfection with 0.2 μg envelope plasmid is slightly lower than that from 48 hours, which is not readily consistent with our early findings in Fig. 4, where early harvest time favors higher infectivity. This lower infectivity at 18 hours was possibly due to the inclusion of excess vector DNA under this condition, where 3.8 μg vector DNA was included together with 0.2 μg envelope plasmid. This result was confirmed in a side-by-side comparison, where 0.2 μg envelope plasmid was transfected in the presence or absence of 3.8 μg vector DNA. Presence of vector DNA indeed generated virions of lower infectivity at 18 hours post transfection. This subtle difference suggests that inclusion of vector DNA may delay the expression of envelope glycoproteins, either through reduction in the uptake of envelope plasmids or a lower expression of envelope mRNA since these vector DNAs do carry Cytomegalovirus promoters by themselves, which may compete for RNA polymerases. The relatively lower infectivity observed at 18 hours in comparison to 48 hours was further supported by the western blots in Fig. 11, where negligible amount of gp120 was produced at 18 hours in comparison to that at 48 hours. Altogether, these results imply that a threshold level of gp120 may be required for HIV virion infectivity. Lower envelope input would require a longer time to accumulate sufficient gp120 expression to produce infectious virions, which explains why the peak values for envelope plasmids is different for 18 versus 48-hour virions. Lastly, for virions collected 48 hours post transfection, we notice a significant reduction in the number of physical particles when envelope plasmid input exceeds 1 μg (Fig. 10C dashed lines). This trend is absent for virions collected at 18 hours, suggesting that there is a potential competition between Gag and envelope in their protein expression at late time points. The level of Gag expression was reduced when excess envelope glycoproteins were produced in response to high plasmid input.

### Virion Infectivity in Rev-CEM Cells

**Figure 12:**
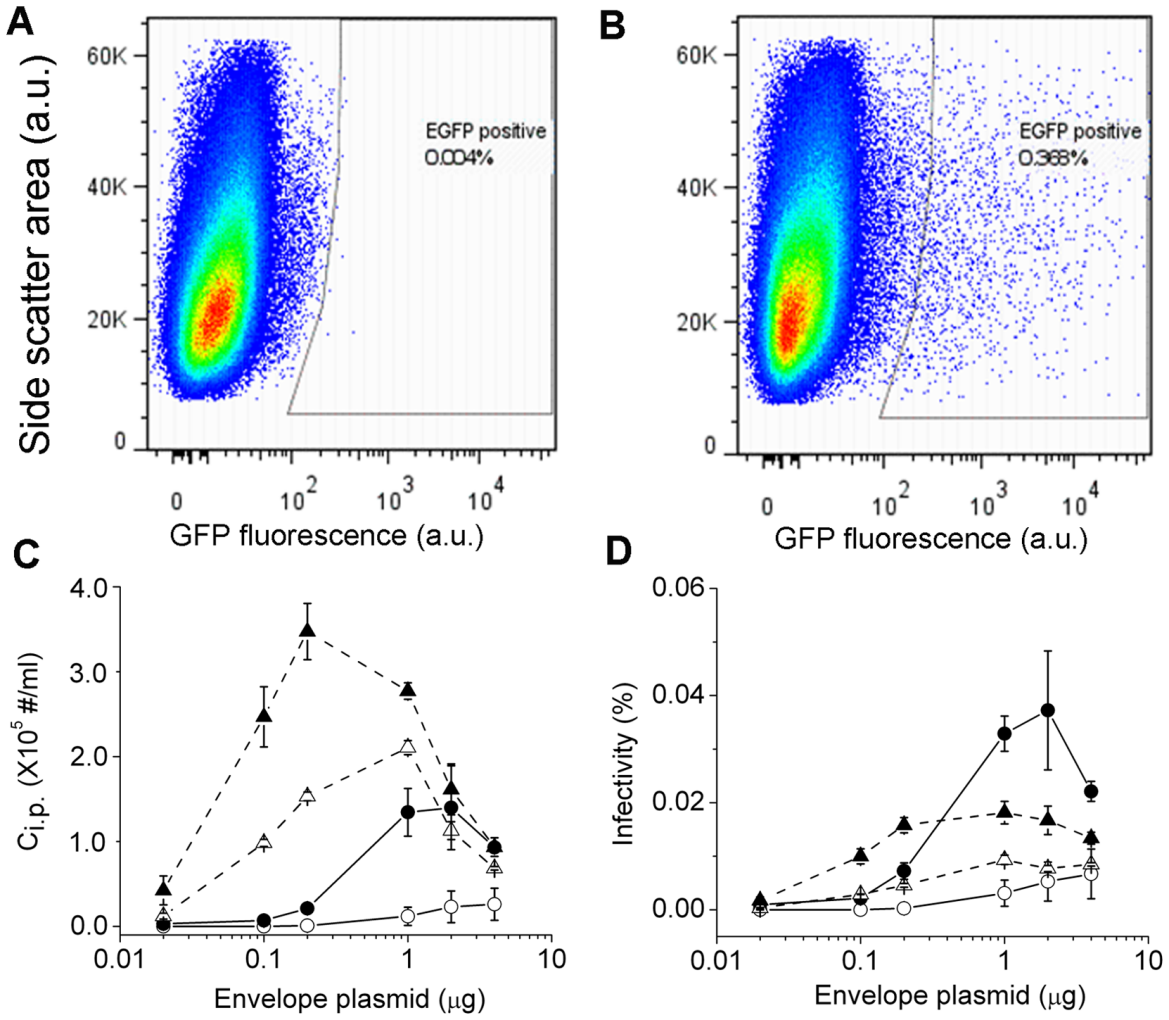
Infection assay in Rev-CEM cells for various single-cycle HIV-1 virions. (A) Negative control in the absence of infection, which was used to gate cell populations in infected cells. (B) A representative cytogram for Rev-CEM cells infected by single-cycle virions generated with 2 μg envelope. The virions were harvested 18 hours post transfection with media change six hours post transfection. (C) and (D) show C_i.p._ and infectivity in Rev-CEM cells for virions produced with various inputs of envelope plasmid pcDNA3.1REC. Circles and triangles are for virions collected 18 and 48 hours post transfection, respectively, with filled symbols representing samples with media change and open symbols representing samples without media change. Error bars are standard deviations from three independent replicates of the same experiments.

The above infection results were obtained for TZM-bl cells, which were engineered [31], [32], [33], [34], [35] to overexpress CD4 and CCR5 coreceptors. To determine how the above results may vary with cell lines, we used the same batches of viruses as described above and infected Rev-CEM cells [55], a CD4^+^ T cell line that has been used as an indicator cell line to quantitate HIV-1 infection. The reporter gene expression in this cell line depends on HIV-1 Rev protein activity so that false positives upon HIV-1 infection are low. Upon productive infection by HIV-1 virions, these cells express GFP which can be quantified using flow cytometry. As shown in Fig. 12A, we use the mock-infected cells to set the gates for quantitation of GFP positive cells and a typical infection result is shown in Fig. 12B. The number of GFP positive cells was linearly dependent on the input virus concentration, which allowed us to measure C_i.p._ based on the slope from a linear regression. The C_i.p._ values from these experiments together with virion infectivity are plotted in Fig. 12C and D as a function of envelope plasmid input, using the same set of lines and symbols as shown in Fig. 10. Consistent with previous findings, conditions that favor higher infectivity in TZM-bl cells also yield higher infectivity in Rev-CEM cells, including virion harvest time, media change post transfection and envelope plasmid input. As a result, the overall shape of these plots is similar (Fig. 10A versus Fig. 12D). These results suggest that these culture conditions work by increasing the intrinsic infectivity of a virus pool, possibly though reduction in the number of defective or less infectious virions. However, the magnitudes of these effects are not as high as the effect of host cells on HIV virion infectivity. Despite the same batches of virions used for infection, the C_i.p._ values from Rev-CEM cells are more than tenfold lower than those from TZM-bl cells (Fig. 12C). As a result, the infectivity in Rev-CEM cells is more than tenfold lower than TZM-bl cells. Even for the most optimized virions (media change, 18 hour harvest post transfection, 2 μg envelope plasmid), the apparent infectivity of virions varies by 19-fold between these two host cells. These results suggest that the difference in viral-cell interactions may pose a major barrier that restricts HIV virion infectivity [30].

## Discussion

The apparent low infectivity of cell-free HIV virions has been a subject of debate. Whether this is due to virions themselves having intrinsically low infectivity or because of viral-cell interactions that restrict HIV-1 infection remains to be elucidated. The assumption behind *intrinsic* low infectivity for these virions is that a large proportion of virion particles are defective, the fraction of which may vary in the viral life cycle. Molecularly cloned HIV-1 that is capable of only a single round of infection [23], [24] offers a unique tool to address these questions. These virions are produced from proviral DNA clones instead of provirus reverse transcribed from viral RNA genome. Mutations in viral proteins due to RT errors or APOBEC3 activity are thus eliminated. Through variation of culture conditions to produce these virions, it may be possible to optimize the intrinsic infectivity of HIV virions, through which to understand the molecular mechanisms that govern the intrinsic infectivity of HIV-1 virions.

As we found, the infectivity of single-cycle HIV-1 virions varies with culture conditions by more than one order of magnitude. Virion harvest time, media change after transfection, and envelope plasmid input can all impact HIV-1 infectivity. These results can be explained by a combination of three different mechanisms: virion spontaneous inactivation, simultaneous production of defective or less infectious particles, and incorporation of envelope glycoprotein into Gag particles. An early harvest time generates virions of higher infectivity. This effect can be as large as fivefold (Fig. 4C). One mechanism behind this phenomenon is the decay of virion infectivity over time as a result of biophysical instability (Fig. 5), which competes with the production of infectious virions. Virions harvested at later times post transfection contain more defective virions, which results in an apparent lower infectivity. However, this is not the only mechanism. Our data suggest that defective or less infectious particles are also produced by the cells together with infectious virions, which lowers the infectivity of a virus pool. When 293T cells are transfected with a mixture of mutant provirus and envelope plasmids, cells that carry the mutant provirus only will produce defective particles, the concentration of which increases with incubation time (Fig. 4B). Cells that carry both plasmids but have low envelope plasmid will produce less infectious particles (Fig. 10A). The presence of these defective or less infectious particles in a virus pool decreases the overall infectivity. More envelope DNA during transfection favors cells that carry both plasmids, and the increased expression of envelope glycoproteins gp120 also leads to an increase of HIV-1 virion infectivity. Lastly, a kinetic difference in envelope and Gag protein availability can also give rise to the time dependence of HIV virion infectivity. For instance, at early time points post transfection, more envelope glycoproteins may be available than Gag proteins, which lead to virions with more envelope incorporation and thus a higher infectivity. As time goes on, more Gag proteins than envelope glycoproteins may be available and thus results in a lower overall infectivity for the virus pool. This mechanism is likely given the fact that Gag can independently bud [56], [57] to form defective particles [23] in the absence of envelope glycoproteins. In the presence of envelope glycoproteins, both ‘passive’ and ‘direct Gag-Env interaction’ models may operate [52]. Thus the level of envelope proteins incorporated into particles may well depend on the level of envelope protein expression [47]. Future studies to quantify the kinetic difference between the expressions of these two proteins can reveal answers to this important question.

Taken together, the overall infectivity of HIV-1 virions can be improved through variation in culture conditions. These optimized culture conditions apply to HIV-1 virions tagged with fluorescent proteins, and increase virion infectivity in CD4^+^ T cell lines, suggesting that these conditions work by reducing defective or less infectious virions and thus increase the overall infectivity of a virus pool. Nevertheless, these improvements are not as dramatic as the impact of host cells on HIV-1 infectivity. When Rev-CEM cells were used, the highest infectivity we could achieve for the most optimized virions was only 0.037%, which is 19-fold lower than that in TZM-bl cells. These results indicate that host cells may pose a major barrier for HIV-1 infectivity even though the intrinsic infectivity of HIV-1 virions can be optimized. As indicated by the results in TZM-bl cells, at least 0.7% of virions are infectious. However, due to differences in host cells, only a small proportion of these virions are able to establish a productive infection in Rev-CEM cells. These results suggest that the viral-cell interactions likely limit the infectivity of HIV-1 virions. In current settings, several mechanisms may potentially contribute to this huge variation of viral infectivity with host cell lines. First, the receptor and coreceptor densities are different for these two cell lines. For TZM-bl cells, it has been estimated that ∼1–5×10^5^ CD4 receptors and ∼2×10^4^ CXCR4 coreceptors are present on a single cell surface [31], [35]. In contrast, 2.4×10^4^ CD4 receptors and ∼2,000 CXCR4 coreceptors may be present on a single Rev-CEM cell surface based on an indirect comparison [55], [58], [59]. The lower expression of CD4 and CXCR4 coreceptor on Rev-CEM may partly contribute to the observed differences in viral infectivity, and future studies are needed to establish the mechanisms behind. Second, difference in post entry steps between these two cell lines may give rise to a difference in viral infectivity. These post entry steps may include not only steps that lead to the provirus integration but also events that lead to the activation and expression of reporter genes, which are the direct readouts in current assays. For all these events, the single-cycle HIV-1 virions are ideal materials to further address these questions because the single replication cycle allows detailed dissection of individual steps in the viral life cycle and their contribution to the overall infectivity of the virion. For instance, the kinetics of virion attachment and internalization can be measured and the impact of these early infection events on the outcome of infection can be quantitated. HIV virions with optimized infectivity can be used in these mechanistic studies to understand the molecular basis of the apparent low infectivity.

## Materials and Methods

### Construction of plasmids

NL4-3 is an HIV-1 strain widely used for production of cloned HIV-1 virions [60], [61], [62]. To produce single-cycle virus, we introduced a frameshift mutation within the envelope coding region of the NL4-3 provirus, which resulted in premature stop codons in the open reading frame (ORF) for the envelope glycoprotein [61]. Cotransfection of the mutant provirus together with envelope expression plasmid in 293T cells allows production of single-cycle virus. Three plasmids, pNL4-3 (cat#114), pEGFP-Vpr (cat#11386), and pNL4-3.Luc3.R^-^E^-^(cat#3418) were obtained through the NIH AIDS Research and Reference Reagent Program. Construction of various other plasmids followed standard molecular biology protocols. Briefly, to construct the provirus DNA that harbors the frameshift mutation in the ORF of the envelope glycoprotein, a 1509-bp DNA fragment between EcoRI and NheI in pNL4-3 was replaced with the corresponding region in pNL4-3.Luc3.R^−^E^−^. The resulting plasmid was designated as pNL4-3E^−^. To construct the envelope glycoprotein expression plasmid, the Rev/Env expression cassette in pNL4-3 was PCR amplified and subcloned into the vector pcDNA3.1(-) using BamHI and XbaI restriction enzymes. The resulting plasmid was designated as pcDNA3.1REC. To construct the provirus clone that carries mutations in both Vpr and envelope glycoproteins, the ORF of Vpr in pNL4-3E^−^ was replaced with that of pNL4-3.Luc3.R^−^E^−^ using two restriction enzymes, PflMI and NheI. The resulting plasmid was designated as pNL4-3R^−^E^−^. To construct the provirus clone that carries enhanced green fluorescent proteins (EGFPs) between matrix and capsid proteins through two linkers that can be cleaved by HIV protease during the virion maturation process [49], three DNA fragments were first PCR-amplified. Fragment 1 uses pNL4-3 as a template, with forward and reverse primers as follows, 5′-GCCAGAGGAGATCTCTCGACG-3′, 5′-CCATCGTACGCTGGAGGTTCTGCACTATAGGGTAATTTTGG-3′. Fragment 2 uses pEGFP-Vpr as a template, with forward and reverse primers as follows, 5′- CTCCAGCGTACGATGGTGAGCAAGGGCGAGGAGC-3′, 5′- CTGGCTCGGCCGCTTGTACAGCTCGTCCATGCCGAGAGTG-3′. Fragment 3 uses pNL4-3 as a template, with forward and reverse primers as follows, 5′- GCGGCCGAGCCAGGTCAGCCAAAATTACCCTATAGTG-3′, 5′- CACTTCCCCTTGGTTCTCTCATC-3′. These three DNA fragments were digested by BssHII/BsiWI, BsiWI/EagI, or EagI/SphI, respectively, and then sequentially ligated and cloned into pNL4-3E^−^ using BssHII and SphI restriction sites. The resulting plasmid was designated as pNL4-3E^-^MA-EGFP-CA. To construct the mCherry expression plasmid pmCherry-Vpr, the ORF of EGFP in pEGFP-Vpr was replaced with that of mCherry using restriction enzymes NheI and HindIII, where the mCherry coding sequence was PCR-amplified from the plasmid H2B-mCherry (Cat#20972, Addgene). All of the plasmids constructed above were subjected to sequencing to confirm their identity before their use in cell culture and transfection procedures.

### Production of single-cycle HIV-1 virions

HEK 293T/17 cells (ATCC, Manassas, VA) were cultured at 37°C with 5% CO_2_ in DMEM supplemented with 10% FBS (HyClone Laboratories, Logan, UT) and penicillin-streptomycin. Throughout, all cell lines were discarded after ten passages and new aliquots of frozen cells were thawed to improve reproducibility of virion production and infection experiments. To produce single-cycle NL4-3E^−^ HIV-1 virions, 10^6^ 293T cells in a 2-ml culture volume were seeded overnight in a 35-mm dish before transfection using the TransIT LT-1 transfection reagent (Mirus Bio, Madison, WI). For each dish, 1 μg of the provirus-containing plasmid pNL4-3E^−^ was used to make the transfection reagent mixture, together with various amount of envelope expression plasmid pcDNA3.1REC as indicated throughout the text. The transfection reagent mixture was incubated at room temperature for 15 min before drop wise addition to the culture media. At designated time points post transfection, the culture media together with the transfection reagents was replaced with fresh complete media and the incubation was continued at 37°C with 5% CO_2_. At various time points post transfection, the entire culture media containing single-cycle HIV-1 viruses was collected and filtered through a 0.45-µm syringe filter (Millex-HV PVDF, Millipore) in less than 10 minutes on average. The filtrate was then aliquoted on ice, flash-frozen in either liquid nitrogen or dry ice/ethanol bath and stored in a −80°C freezer. Control experiments showed that virion infectivity remains constant during incubation on ice for up to six hours and flash-freezing in complete media leads to no loss of virion infectivity. To produce single-cycle virions tagged with EGFP, the same procedures were used as described above except the input DNA used during transfection. In detail, HIV virions tagged with EGFP- Vpr fusion proteins [48] were generated by transfection of 293T cells with 1.0 μg pNL4-3R^−^E^−^ plasmid, 0.1 μg pcDNA3.1REC and 0.3 μg pEGFP-Vpr in 2-ml culture volume in a 35-mm dish. 0.3 μg pEGFP-Vpr per dish was chosen based on the dependence of virion infectivity on pEGFP-Vpr input (Fig. 8A), which showed intermediate values among all the DNA concentrations tested. HIV virions carrying free EGFPs were generated by transfection of 293T cells with 0.5 μg pNL4-3E^-^ plasmid, 0.5 μg pNL4-3E^-^MA-EGFP-CA plasmid and 0.1 μg pcDNA3.1REC in 2-ml volume in a 35-mm dish. The 1:1 mixing ratio between the untagged and tagged provirus DNA was chosen based on the dependence of virion infectivity on the ratio between these two plasmids (Fig. 8B), which showed intermediate values among all the input ratios tested. As reported in literature, the typical production of HIV-1 virions using 293T cells involves calcium phosphate transfection [62]. Our comparison showed that the titer of HIV-1 virions produced by Mirus LT-1 transfection is on average 12-fold higher than that from calcium phosphate transfection. Thus, we have used Mirus LT-1 transfection reagents throughout.

### Infection assay in TZM-bl cell line

TZM-bl cells (cat#8129, NIH AIDS Research and Reference Reagent Program) were cultured at 37°C with 5% CO_2_ in DMEM supplemented with 10% FBS and penicillin-streptomycin. To determine the concentration of infectious HIV-1 particles, C_i.p._, 8×10^4^ TZM-bl cells in a 1-ml culture volume were seeded in each well of a 12-well plate one day prior to infection. On the next day, virus stocks taken out of −80°C freezer were placed in a room temperature water bath until just thawed, and serially diluted in complete media containing 20 μg/ml DEAE-dextran. 100 μl of viruses at each dilution were layered on top of the cell and the infection was continued for two hours at 37°C with gentle rocking every 30 min. At the end of two hours, 1 ml of complete media was added to each well and the incubation was continued at 37°C for 48 hours with 5% CO_2_. At the end of 48 hours, cells were fixed in 2% gluteraldehyde at room temperature for five minutes. After fixation, the cells were washed three times with PBS, and stained for 50 min at 37°C using cell staining solution provided in the beta-galactosidase staining kit (Mirus Bio, Madison, WI). After incubation, the cells were washed three times with milliQ water and the number of blue cells in each well was counted with a Nikon TS100-F inverted microscope. For luciferase activity assay to monitor HIV-1 infection, luciferase expression in TZM-bl cells upon viral infection was measured using Bright-Glo^TM^ Luciferase Assay System (Promega) following manufacturer’s instructions. Briefly, 48 hours after infection, culture media was removed and TZM-bl cells were washed with Dulbecco's Phosphate-Buffered Saline (DPBS). 200 μl of Glo lysis buffer were then added to each well. The cells were incubated for five minutes at room temperature to allow cell lysis. At the end of five minutes, 50 μl lysate from each well was transferred to a single well in a 96-well black microtiter plate (Costar). 50 μl of Bright-Glo^TM^ assay reagent was then added to each well and mixed. Luminescence was measured using Synergy^TM^ HT multi-mode plate reader (BioTek Instruments Inc., Vermont) and background luminescence was subtracted using TZM-bl cells without virus infection.

### P24 ELISA Assay

To determine the concentration of physical particles for each virion preparations, C_p.p._, we measured p24 values for each virion prep using p24 ELISA and converted p24 values to concentrations of physical particles. The p24 values were measured using HIV-1 p24 Antigen Capture Kit (Advanced Bioscience Laboratories, Rockville, MD) following the manufacturer's instructions. Briefly, properly diluted virus samples were lysed and captured in a micro-ELISA plate at 37°C for one hour. The wells were then washed and the specifically captured p24 antigen was incubated with human anti-p24 polyclonal antibodies conjugated with peroxidase at 37°C for one hour. At the end of incubation, peroxidase substrate was added and the reaction was continued for 30 minutes at room temperature. The reaction was stopped by adding 2N Sulfuric acid. The absorbance at 450 nm was measured using a Synergy^TM^ HT multi-mode microplate reader. The stoichiometry of Gag protein in HIV-1 has been determined to be approximately 2,500 [45], [46]. Using a molecular weight of 24 kD for p24, this yields 1×10^7^ particles per ng of p24. Thus one can estimate the number of physical particles based on p24 ELISA measurement for each virion preparation. Independently, we also validated this method using confocal imaging and direct counting of virion particles (Fig. 3). The infectivity of single-cycle HIV-1 was calculated by taking the ratio between infectious particle concentration (C_i.p._) and physical particle concentration (C_p.p._). Throughout, the error bars we have presented are standard deviations from replicates of the same experiments unless otherwise noted.

### Confocal imaging and counting of virions

HIV Virions carrying EGFP-Vpr at various dilutions in 200-μl complete media (90% DMEM with 10% FBS) were deposited onto a Poly-L-Lysine- coated coverslip, and incubated at room temperature for 30 minutes [63]. The media was then removed and virions on the surface were fixed with 4% paraformaldehyde (PFA) for 10 minutes at room temperature. The coverslip was then washed with PBS and mounted onto a glass cover slide with 3 µl mounting media, sealed with nail polish and imaged using an Olympus FluoView 500 Laser Scanning Confocal Microscope. The virion particles in a fluorescence image were identified and quantitated using a custom-written MATLAB script. In this script, the virion particles were identified based on a global threshold automatically established by maximizing the inter-class variance between the foreground and background (Otsu's method) [64], which eliminates the use of any subjective parameters during image analysis. At least ten different areas on a single coverslip sample were imaged and the resulting particle numbers in each area were averaged and plotted in Fig. 3C.

### Western blotting

Samples for western blotting were collected from transfected 293T cell culture as described above. For viruses collected from culture media, they were directly mixed with 6× SDS-PAGE loading buffer containing β-mercaptoethanol and incubated at 95°C for five minutes before loading onto a gel. For cell lysate samples, 293T cells were first washed with cold PBS, and then lysed in 200 μl of radioimmunoprecipitation assay (RIPA) buffer (50 mM Tris•Cl, 150 mM NaCl, 1% Triton X-100, 0.5% deoxycholate, and 0.1% SDS, pH 7.5) containing protease inhibitor cocktails (Thermo Scientific) for 15 minutes on ice. Cell lysate was clarified by centrifugation at 12,000 g for 20 minutes at 4°C. The supernatants were then mixed with 6× SDS-PAGE loading buffer containing β-mercaptoethanol and incubated at 95°C for five minutes. Both virus and cell lysate samples were subjected to a 4–15% gradient gel (mini-protean TGX, Bio-Rad). The resolved protein bands were electrotransferred onto supported nitrocellulose membranes and further probed with antibodies. Primary antibodies used for detection were as follows: sheep anti-gp120 polyserum (Cat#288, NIH AIDS reagent program), and mouse anti-beta actin (Sigma), which were diluted at 1:5,000, and 1:1,000, respectively. The secondary antibodies were as follows: anti-sheep alkaline phosphatase (AP)-conjugated secondary antibody (Sigma), and anti-mouse AP-conjugated secondary antibody (Sigma), which were diluted at 1:30,000, and 1:10,000, respectively. Protein bands were developed with the Nitroblue tetrazolium chloride/ 5-bromo-4-chloro-3'-indolyphosphate p-toluidine salt substrates (NBT/BCIP, Roche) in a buffer containing 0.1 M Tris•HCl, 0.1 M NaCl, and 0.05 M MgCl_2_ at pH 9.5. The protein bands were then scanned with a desktop scanner (Dell) and quantified using ImageJ (NIH, Bethesda, USA, http://imagej.nih.gov/ij/).

### Infection assay in Rev-CEM cell line

Rev-CEM cells (cat#11467, NIH AIDS Research and Reference Reagent Program) were cultured at 37°C with 5% CO_2_ in RPMI supplemented with 10% FBS and penicillin-streptomycin. A 24-well plate was used to determine C_i.p._ of HIV-1 virions in Rev-CEM cells. For each well, 100 μl of viruses serially diluted in complete media were mixed with 2×10^5^ Rev-CEM cells in a total volume of 300 μl containing 5 μg/ml DEAE-dextran. The plate was gently rocked every 15 minutes for the initial two hours of incubation at 37°C. At the end of two hours, 1.7 ml of complete media was added to each well and the incubation was continued at 37°C with 5% CO_2_ for five days. At the end of the incubation, cells were fixed with 2% PFA for five minutes at room temperature. The cells were then washed with PBS and quantitated with a flow cytometer (iCyt Synergy, Sony) equipped with a 488 nm laser. GFP emission was monitored using a 50 nm band pass filter centered at 525 nm.
